# *N*-Heterocyclic carbene–carbodiimide (NHC–CDI) betaine adducts: synthesis, characterization, properties, and applications

**DOI:** 10.1039/d0sc06465c

**Published:** 2021-01-19

**Authors:** Jessica R. Lamb, Christopher M. Brown, Jeremiah A. Johnson

**Affiliations:** Department of Chemistry, Massachusetts Institute of Technology 77 Massachusetts Avenue Cambridge Massachusetts 02139 USA jrlamb@umn.edu jaj2109@mit.edu

## Abstract

*N*-Heterocyclic carbenes (NHCs) are an important class of reactive organic molecules used as ligands, organocatalysts, and σ-donors in a variety of electroneutral ylide or betaine adducts with main-group compounds. An emerging class of betaine adducts made from the reaction of NHCs with carbodiimides (CDIs) form zwitterionic amidinate-like structures with tunable properties based on the highly modular NHC and CDI scaffolds. The adduct stability is controlled by the substituents on the CDI nitrogens, while the NHC substituents greatly affect the configuration of the adduct in the solid state. This *Perspective* is intended as a primer to these adducts, touching on their history, synthesis, characterization, and general properties. Despite the infancy of the field, NHC–CDI adducts have been applied as amidinate-type ligands for transition metals and nanoparticles, as junctions in zwitterionic polymers, and to stabilize distonic radical cations. These applications and potential future directions are discussed.

## Abstract 2

*N*-heterocyclic carbene-carbodiimide betaine adducts are zwitterionic amidinate-like structures with tunable properties that have applications as ligands, junctions in supramolecular polymers, and stabilizers for radical cations.

## Graphical abstract

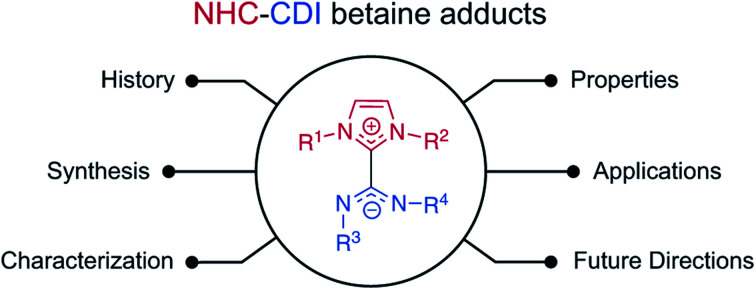

## 1. Introduction

**Fig. 1:**
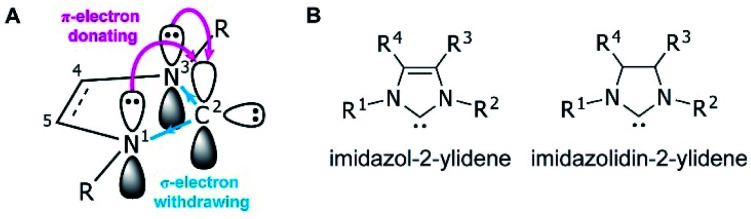
(A) Ground-state electronic structure of imidazol-2-ylidenes with numbering at the heterocycle shown. (B) NHCs of the imidazolidium and imidazolidinium based classes, with unsaturated and saturated backbones, respectively.

Since the first demonstrations of persistent carbenes,^1,2^*N*-heterocyclic carbenes (NHCs) have been used extensively in transition metal and main group chemistries,^3–5^ as organocatalysts,^6^ as ligands in organometallic catalysts,^7^ and in materials chemistry.^8,9^ NHCs are broadly defined as heterocyclic compounds that contain a divalent carbon and at least one nitrogen within the ring.^10^ They act as neutral, two-electron species, and are frequently used as phosphine analogues, though with stronger metal–ligand bonds due to their higher σ-donating abilities.^11^ Kinetic stability is aided by bulky substituents adjacent to the carbene, while the nitrogen substituent(s) electronically stabilize the empty carbene orbital through resonance^12^ (Fig. 1A).

NHCs are extremely modular, allowing facile tuning of their electronic and steric properties. Perhaps the most well-studied are the five-membered imidazolium- and imidazolidinium-based NHCs (Fig. 1B).^11,13^ Variations can be made at the nitrogen substituents (*i.e.*, the wingtip positions) to make either symmetric (R^1^ = R^2^) or asymmetric (R^1^ ≠ R^2^) species, and likewise the 4- and 5-positions (R^3^ and R^4^) can be modified, though with typically a lesser effect on the electronics of the system.^13^ Consequently, chemists have access to a huge synthetic library of cationic heterocycles to use as NHC precursors.^14^

**Fig. 2:**
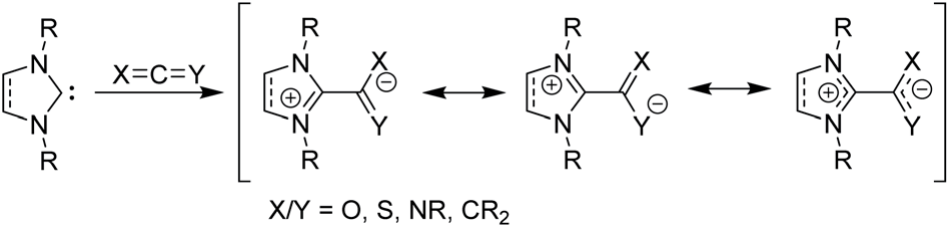
Zwitterionic betaine adducts made from *N*-heterocyclic carbenes with allenes, ketenes, or heteroallenes.

Beyond their traditional applications as ligands and organocatalysts, NHCs can add to or insert themselves into a variety of organic species to form stable, neutral adducts, and if a suitable electrophile is used, zwitterionic adducts can be formed.^15^ Halogens or boranes lead to the formation of ylides, while reactions with allenes, ketenes, or heteroallenes give betaine adducts, which can only be represented by resonance forms with formal charges (Fig. 2). To date, many stable, crystalline NHC–betaine adducts with CS_2_, CO_2_, isothiocyanates, and isocyanates have been reported and reviewed elsewhere.^16^

In this *Perspective*, we focus on betaine adducts made from NHCs and carbodiimides (CDIs), *i.e.*, zwitterionic amidinates (NHC–CDIs, Fig. 2 X and Y = NR). Amidinates are considered the nitrogen analogues of carboxylates.^17^ They act as coordinating ligands and feature a wide range of coordination modes; many amidinate complexes of transition metals,^18–20^ lanthanides,^17^ actinides,^21^ and main group elements^22^ have been reported. One important difference compared to carboxylate ligands is that the nitrogen atoms in amidinates nearly always carry an additional substituent, which allows for fine-tuning of the electronic and steric properties of the ligand system.^22^ Combined with the similarly modular NHC framework, this feature presents a unique opportunity for tailoring NHC–CDI adducts for specific applications. NHCs and amidinates are also typically both air- and moisture-sensitive, whereas NHC–CDI adducts made from *N*,*N*′-diaryl CDIs result in net neutral compounds that are more tolerant to ambient conditions while retaining a strongly Lewis basic character at the amidinyl nitrogens.^23^ This *Perspective* will introduce the history, common synthetic and characterization methods, and a summary of the structure–property relationships of the currently known NHC–CDI adducts. Finally, applications to coordination chemistry, nanoparticle functionalization, supramolecular polymers, and distonic radical cation stabilization will be summarized along with future directions of the field.

## 2. History

To our knowledge, the first zwitterionic NHC–CDI adduct was proposed by Takamizawa and co-workers in 1974.^24^ These authors found that the addition of a thiazolium iodide species to an *N*,*N*′-diaryl CDI in the presence of triethylamine (NEt_3_) yields a mixture containing a cationic thiazolium–CDI adduct and a spirocycle made *via* cycloaddition with a second equivalent of CDI. It was proposed that deprotonation of the thiazolium adduct leads to a zwitterionic NHC–CDI intermediate, which can then react with another CDI to give the cycloadduct. While the zwitterionic intermediate could not be isolated, it was noted that analogous zwitterionic adducts formed from the reaction of a phosphorus ylide with an *N*,*N*′-diaryl CDI had been previously isolated.^25^

It was not until 25 years later that the first stable, isolable NHC–CDI was reported by Kuhn and co-workers.^26^ 1,3-Diisopropyl-4,5-dimethylimidazol-2-ylidene was allowed to react with diisopropylcarbodiimide, yielding a zwitterionic adduct which was characterized by NMR spectroscopy and X-ray crystallography. The strong Brønsted basicity of the amidinate nitrogen atoms caused decomposition of the adduct upon exposure to moisture, but also indicated the potential for NHC–CDIs as ligands for metallic systems or as organic bases, while remaining electroneutral. Despite this promise, the next report on isolated NHC–CDI adducts did not come, to our knowledge, until bench-stable variants utilizing *N*,*N*′-diaryl CDIs were discovered simultaneously by the Johnson and Cámpora groups in 2015.^23,27^ Since then, the facile synthesis, air- and moisture-stability, and interesting properties of these betaine adducts have spurred new interest in the field.

## 3. Synthesis and structure

### 3.1 Methods for NHC–CDI synthesis

#### 3.1.1 Imidazol-2-ylidene and imidazolidin-2-ylidene

**Fig. 3:**
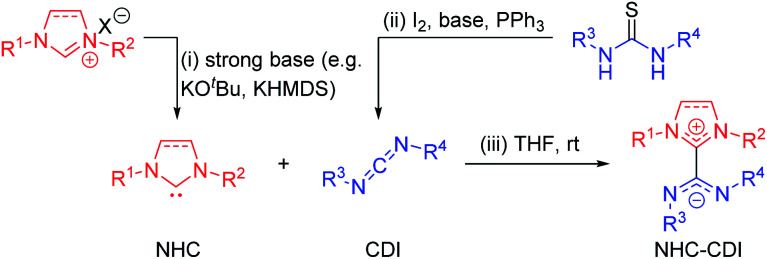
Common synthetic routes to (i) free NHC by deprotonation of an imidazolium salt precursor, (ii) free CDI by desulfurization of a thiourea, and (iii) NHC–CDI adduct.

A variety of free NHCs and NHC precursors are available commercially as well as through robust synthetic methods,^14,28^ which have been reviewed elsewhere.^14^ Thus far, NHC–CDI adducts have utilized imidazol-2-ylidene and imidazolidin-2-ylidene NHCs (Fig. 1B). The free carbenes are most typically synthesized *via* deprotonation of the corresponding imidazolium or imidazolidinium salt by a strong base, such as sodium hydride,^29^ potassium *tert*-butoxide (KO^*t*^Bu),^30^*n*-butyl lithium (^*n*^BuLi),^31^ potassium metal,^32^ or potassium hexamethyldisilazide (KHMDS)^23,33^ (Fig. 3i). The salt byproduct is often, but not always, removed from the free NHC by filtration before subsequent reactions to avoid complications that arise from the presence of residual base or their conjugate acid.

To avoid the use of very strong bases, light-triggered deprotonation has been achieved using isopropylthioxanthone (ITX) with an azolium salt precursor containing a tetraphenylborate (BPh_4_^−^) counteranion.^34^ Photoexcitation of ITX results in a triplet excited state that can accept a single electron from BPh_4_^−^ to form ITX˙^−^ which abstracts a proton from the azolium cation to generate the free carbene and ITX–H˙. Heat-sensitive progenitors can also produce free NHCs without the use of a strong base. This strategy is common for latent organocatalysis^35^ and has been utilized for the synthesis of NHC–CDI adducts by Johnson and co-workers, who first used carboxylate and diphenylphosphine adducts of SIMes^23^ and later synthesized C_6_F_5_-masked NHCs by exposing the corresponding diamines to pentafluorobenzaldehyde in acetic acid at room temperature.^33^

#### 3.1.2 Carbodiimides

Commercially available CDIs are most commonly used as amide coupling reagents or precursors to a variety of nitrogen-containing heterocycles.^36^ With the exception of commercial di(*para*-tolyl)carbodiimide (CDI^*p*Tol^) and dicyclohexylcarbodiimide (CDI^Cy^), CDIs used to make NHC–CDI adducts have been most commonly synthesized from the corresponding (thio)urea compounds, which in turn can be made from nucleophilic attack of an amine on an iso(thio)cyanate.^33,37^ Desulfurization of thioureas is accomplished with thiophilic iodine and a base, such as triethylamine^38^ or dimethylaminopyridine (DMAP)^37^ (Fig. 3ii). Triphenylphosphine can be added to increase the efficiency of this reaction^33^ through its complexation with iodine.^39,40^ Ureas can be converted to carbodiimides in a similar fashion upon the addition of triethylamine, carbon tetrabromide, and triphenylphosphine.^33^

#### 3.1.3 NHC–CDIs

Johnson and co-workers serendipitously discovered that *N*,*N*′-diaryl CDIs form stable adducts with NHCs upon heating SIMes or its derivatives for extended periods of time in toluene or dichlorobenzene at >100 °C.^23^ Mechanistically, they proposed a concerted [3 + 2] cycloelimination of the NHC to generate ethylene and the corresponding *N*,*N*′-diaryl CDI followed by trapping with another equivalent of NHC. Though this method led to the discovery of stable NHC–CDI adducts, it is limited by the need for long reaction times and the necessity that the NHC and CDI substituents must be the same.

The most common method for the preparation of NHC–CDI adducts involves directly adding a free carbene to a free CDI in an appropriate solvent (Fig. 3iii).^23,27,30,33,41,42^ The often brightly-colored products form quickly at room temperature and can in many cases be readily purified by washing with, *e.g.*, cold hexanes. If a thermally-labile NHC precursor is used instead of a free carbene, the masked NHC and free CDI can be heated in an appropriate solvent to form the NHC–CDI adduct.^33^ It is important to emphasize that the method of preparation is largely personal preference, as any method for releasing a free NHC in the presence of a free CDI will likely result in the formation of the same adduct.

**Fig. 4:**
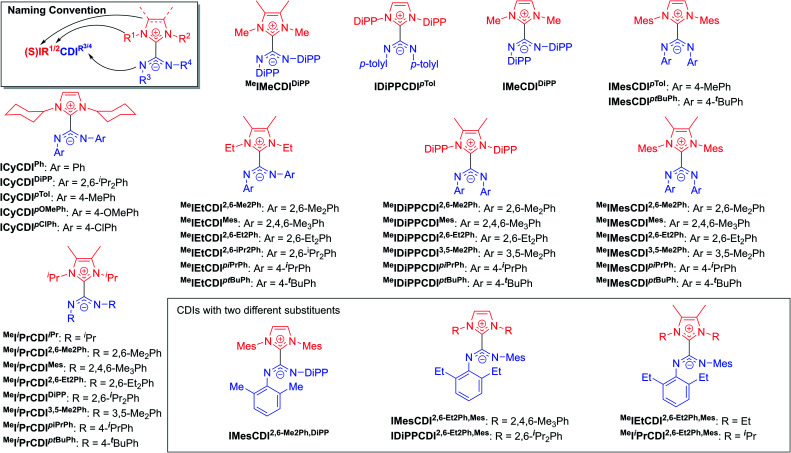
Previously synthesized imidazol-2-ylidene based NHC–CDI adducts.

**Fig. 5:**
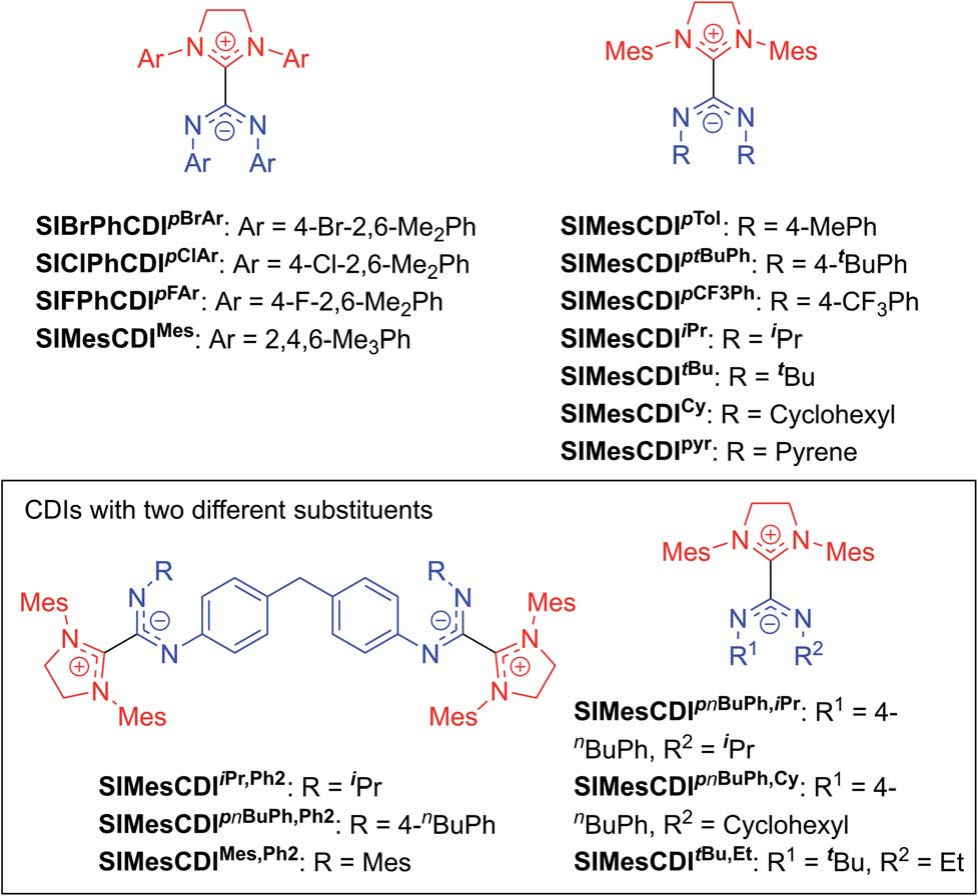
Previously synthesized imidazolidin-2-ylidene based discrete NHC–CDI adducts.

The highly modular nature of both the NHC and the CDI fragments have led to the synthesis of a wide range of NHC–CDI adducts. Previously synthesized imidazol-2-ylidene-based adducts are shown in Fig. 4 and imidazolidin-2-ylidene-based adducts are shown in Fig. 5. The naming convention consists of the NHC backbone (I for imidazol-2-ylidene, ^Me^I for 4,5-dimethylimidazol-2-ylidene, or SI for saturated imidazolidin-2-ylidene) followed by the NHC wingtip substituents, “CDI”, and the amidinate substituents as superscripts.

Cámpora and co-workers synthesized a variety of imidazol-2-ylidene-based adducts with alkyl substituents (methyls and cyclohexyls) in the wingtip positions.^27,30,41,42^ Nembenna and co-workers expanded the library utilizing both *N*,*N*′-dialkyl and *N*,*N*′-diaryl NHCs along with *N*,*N*′-diaryl CDIs with either the same or different substituents.^43^ Variants using saturated NHCs have been made by Johnson and co-workers with *N*,*N*′-diaryl, *N*-aryl-*N*′-alkyl, and *N*,*N*′-dialkyl CDIs, with variable stability (see Section 3.3.1 for more details).^23,33,44^ Additional adducts were made as repeat units of zwitterionic polymers, which will be discussed further in Section 4.2.

### 3.2 Characterization of NHC–CDIs

#### 3.2.1 Nuclear magnetic resonance (NMR) spectroscopy

NMR spectroscopy offers a convenient tool to monitor NHC–CDI formation. For example, the ^1^H NMR resonances of the CDI substituents typically shift upfield upon addition of a free NHC and NHC–CDI formation due to the increased shielding imparted by the negatively charged amidinate region.^33^ The ^13^C{^1^H} NMR resonance of the carbenic carbon has been shown to be a good indicator of NHC–CDI formation. For unsaturated (imidazol-2-ylidene) NHCs, the carbenic carbon peak shifts from 205–220 ppm in the free NHC to 140–152 ppm in the NHC–CDI adduct.^43^ For saturated (imidazolidin-2-ylidene) NHCs, the carbenic carbon is more deshielded with the free carbene and NHC–CDI resonances appearing around 241–245 ppm and 164–166 ppm, respectively.^23,44^

**Fig. 6:**
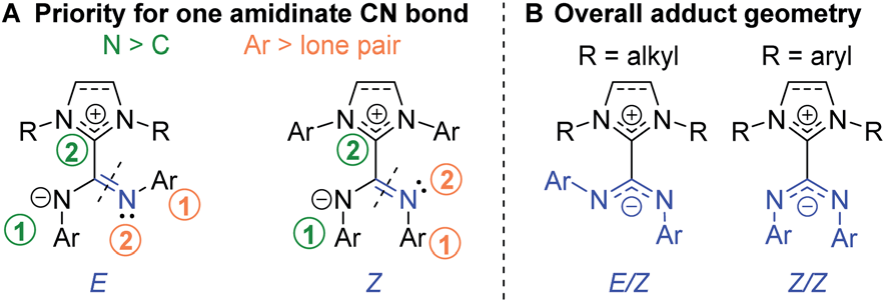
(A) Priorities for assigning the geometry of amidinate C=N bonds and (B) overall geometry for NHC–CDI adducts.

Because of the delocalized double bond character, there is restricted rotation about both amidinate C <svg xmlns="http://www.w3.org/2000/svg" version="1.0" width="23.000000pt" height="16.000000pt" viewBox="0 0 23.000000 16.000000" preserveAspectRatio="xMidYMid meet"><metadata> Created by potrace 1.16, written by Peter Selinger 2001-2019 </metadata><g transform="translate(1.000000,15.000000) scale(0.017500,-0.017500)" fill="currentColor" stroke="none"><path d="M0 440 l0 -40 600 0 600 0 0 40 0 40 -600 0 -600 0 0 -40z M0 280 l0 -40 120 0 120 0 0 40 0 40 -120 0 -120 0 0 -40z M400 280 l0 -40 200 0 200 0 0 40 0 40 -200 0 -200 0 0 -40z M960 280 l0 -40 120 0 120 0 0 40 0 40 -120 0 -120 0 0 -40z"/></g></svg> N bonds of NHC–CDI adducts, orienting the geometry of each bond as transoid (*E*) or cisoid (*Z*) with respect to the other amidinate nitrogen (Fig. 6A). ^15^N cross polarization magic-angle spinning (CP-MAS) solid-state NMR spectroscopy of ICyCDI^Ph^ with one ^15^N-labeled amidinate nitrogen exhibited two signals at 211 and 218 ppm with approximately equal intensity.^30^ Density functional theory (DFT) calculations support that these signals correspond to the two non-equivalent nitrogen atoms of the *E*/*Z* configuration (Fig. 6B). Many ^13^C CP-MAS NMR signals are also duplicated due to the non-equivalent phenyl substituents in the *E*/*Z* configuration. In contrast, solution-state ^1^H and ^13^C{^1^H} NMR spectra show a single set of aromatic resonances, indicating fast exchange between the *E*/*Z* and *Z*/*E* configurations in solution. DFT calculations have suggested that the *E*/*E* geometry is less stable than the *E*/*Z* configuration by 10.2 kcal mol^−1^, ruling out facile observation of the *E*/*E* isomer.^30^

Rotation of the amidinate substituents is highly dependent on the steric bulk of the NHC wingtip substituents. For example, the diastereotopic methyl groups of 2,6-diisopropylphenyl (DiPP) moieties are well-resolved in both the ^1^H and ^13^C{^1^H} NMR spectra for ICyCDI^DiPP^, indicating a locked configuration on the NMR timescale, but the analogous protons for IMeCDI^DiPP^ show a single resonance in the ^1^H NMR spectrum and two broad resonances in ^13^C{^1^H} NMR spectrum, indicating restricted rotation.^41^ The NMR spectra of adducts synthesized from CDIs with two different substituents^33,43^ do not show two sets of peaks for the NHC substituents, indicating that the imidazoyl and amidinyl moieties are effectively orthogonal under the experimental conditions, such that the lack of symmetry of the CDI does not break the symmetry of the NHC.

#### 3.2.2 Single crystal X-ray diffraction (XRD)

Single crystal XRD has been used to study the structures of NHC–CDI adducts in the solid state. The NHC–CDI adducts studied in this way were very soluble in dichloromethane (DCM) and partially soluble in toluene, THF, and hydrocarbons. Therefore, single crystals were generally grown from mixtures of DCM and hexanes,^23,33,43^ though toluene^23^ and acetonitrile (MeCN)^44^ have also been used.

A remarkable feature revealed by the reported crystal structures of NHC–CDIs is the invariability of the core NHC–CDI C–C and C–N bond lengths. Regardless of the steric or electronic properties of the NHC or amidinate substituents, the reported central C–C bond lengths range from only 1.50–1.52 Å. The NHC CN bond lengths are generally 1.34–1.35 Å for unsaturated imidazol-2-ylidene derivatives^41,43^ and slightly shorter at 1.32–1.33 Å for saturated imidazolidin-2-ylidene derivatives.^23,45^ The amidinate CN bond lengths are also fairly uniform for all reported structures at 1.30–1.33 Å. These carbon–nitrogen bond lengths are consistent with delocalized double bonds across the anionic amidinate and cationic carbenic regions of the molecules.

The NHC NCN bond angle is based on the class of NHC used, with unsaturated imidazol-2-ylidenes and saturated imidazolidin-2-ylidenes displaying 106.7–108.3° (ref. 41 and 43) and 111.0–112.2° (ref. 23, 33 and 45) angles, respectively. The amidinate NCN bond angles vary significantly more on the basis of both the class of NHC and the identity of the amidinyl substituents. For imidazol-2-ylidene-based adducts, CDI^DiPP^ yielded amidinate NCN angles of 126.5–127.5°,^41,43^ bis(dialkylphenyl)carbodiimides (dialkylphenyl = 2,6-dimethylphenyl, 3,5-dimethylphenyl, and 2,6-diethylphenyl/mesityl) displayed amidinate angles of 139.0–140.7°,^43^ and CDI^*p*Tol^ had amidinate angles of 130.1°, 138.4°, and 141.6° with ICy,^41^ IMes,^23^ and IDiPP,^23^ respectively. For imidazolidin-2-ylidenes, CDI^Mes^ and derivatives (methyl, bromo, chloro, or fluoro in the *para*-position) showed amidinate angles of 139.0–140.0°.^23^ The solid-state structure of SIMesCDI^pyr^ shows two conformations: one with *C*_2_ symmetry and one distorted from *C*_2_ symmetry, which show amidinate angles of 136.8° and 137.9°, respectively.^45^ The dihedral angles between the NHC and amidinate fragments of the adducts vary wildly from 50.2–87.8°, without obvious correlations to either the steric or electronic properties of the nitrogen substituents.

The adduct geometry seems to be dictated by the NHC wingtip substituents, with *N*,*N*′-dialkyl NHCs leading to an *E*/*Z* configuration^41,43^ and *N*,*N*′-diaryl NHCs yielding a *Z*/*Z* configuration^23,33,43,45^ (Fig. 6B). Notably, ^Me^I^i^PrCDI^2,6-Me2Ph^ displays a *Z*/*Z* geometry despite having isopropyl substituents in the wingtip positions.^43^

#### 3.2.3 UV-vis absorption spectroscopy

Perhaps the most obvious indicator of NHC–CDI formation is the color change to bright yellow/orange that is characteristic for NHC–CDI adducts with *N*,*N*′-diaryl amidinates. UV-vis absorption spectroscopy captures this change *via* a trailing absorbance into the visible region (*λ* > 400 nm).^33^ Notably, the *N*,*N*′,*N*′′,*N*′′′-tetraalkyl NHC–CDI ^Me^I^i^PrCDI^iPr^ synthesized by Kuhn and co-workers is colorless,^26^ suggesting that aryl substituents on the amidinate play a key role in determining the absorption spectrum. TD-DFT calculations suggested that the color corresponds to a HOMO–LUMO transition, where the highest occupied molecular orbital (HOMO) consists of a π-orbital mainly on the anionic amidinate moiety and the lowest unoccupied molecular orbital (LUMO) is mainly centered on the cationic heterocyclic fragment.^41^ The energy of this transition is lower (and therefore in the visible region) because the HOMO is destabilized by nonbonding interactions with filled π-orbitals of the aryl substituents.

#### 3.2.4 Fourier transform infrared (FTIR) spectroscopy

FTIR spectroscopy can be used to monitor the disappearance of the characteristic CDI vibration at 2110–2140 cm^−1^ or the appearance of the amidinate stretching band, which has been reported at 1500–1550 cm^−1^ for imidazolium-based adducts^41^ and slightly higher at 1540–1560 cm^−1^ for imidazolidinium-based adducts.^33^ The NHC C=N stretching modes and C–H flexion modes also overlap near 1500 cm^−1^. An additional asymmetric amidinate NCN stretch can be detected in some systems as a medium-intensity band at ∼1600 cm^−1^.^41^

#### 3.2.5 Other characterization methods

Elemental analysis (EA) and electrospray ionization mass spectrometry (ESI-MS) have also been used to confirm the composition of various NHC–CDI adducts.^33,41,43^ Fluorescence has been detected in the violet region (389–422 nm) for *N*,*N*′,*N*′′,*N*′′′-tetraaryl NHC–CDIs due to conjugation between the amidinate and NHC components, which is supported by DFT calculations of the HOMO and LUMO of an *N*,*N*′,*N*′′,*N*′′′-tetraaryl NHC–CDI.^23^

### 3.3 Properties

#### 3.3.1 Bonding

The carbon–carbon bond between the NHC and the CDI fragments of NHC–CDI adducts is best described as a dative covalent bond in which the NHC lone pair is donated to the central carbon of the CDI. Therefore, these bonds can be viewed as an organic analog to a dative ligand–metal bond and the adduct formation can be described by an equilibrium constant (*K*_eq_).^33^*K*_eq_ values for NHC–CDI adduct formation reported so far are mostly dictated by the electronic and steric properties of the amidinate fragment, as seen by the fact that *N*,*N*′-diaryl CDIs can form air-stable adducts with both *N*,*N*′-dialkyl and *N*,*N*′-diaryl NHCs, but *N*,*N*′-dialkyl CDIs do not form air-stable adducts with either. The improved stability of betaine adducts produced from *N*,*N*′-diaryl CDIs may be from greater delocalization of the amidinate negative charge, as well as the greater electronegativity of sp^2^ over sp^3^ carbons.^23^

**Fig. 7:**
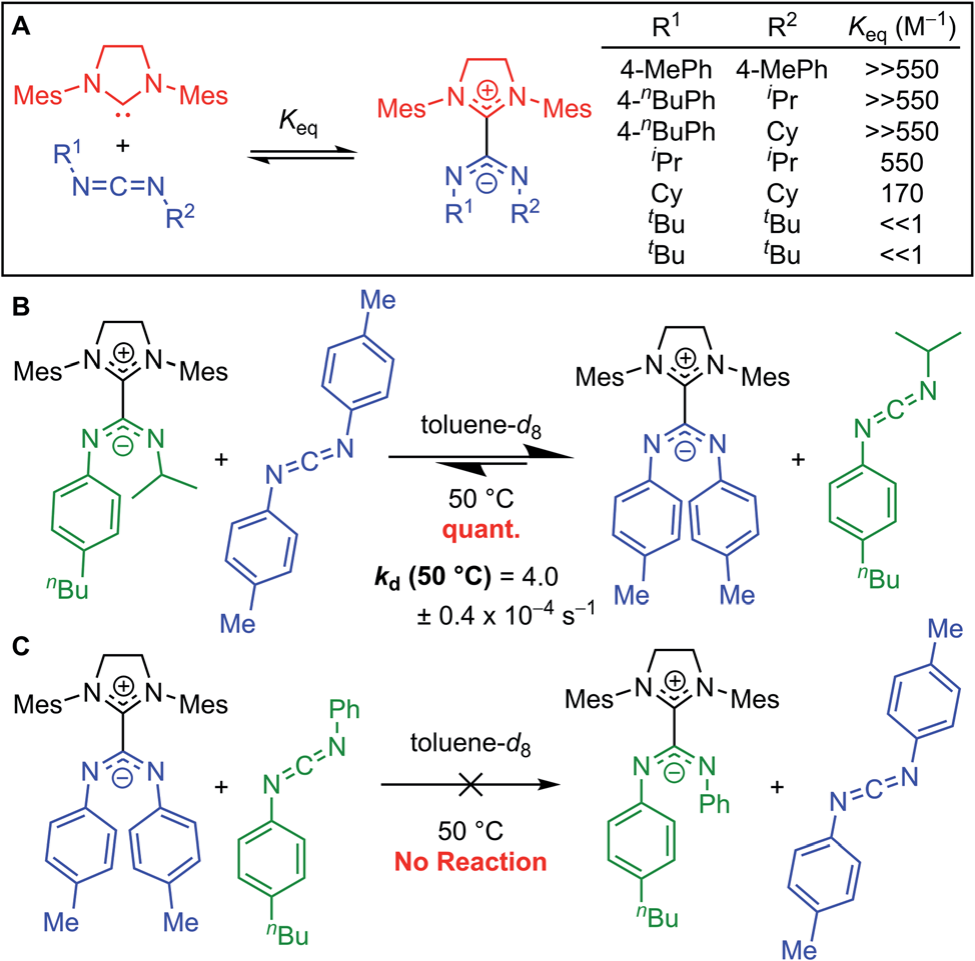
(A) Comparison of binding equilibria of SIMes with *N*,*N*′-diaryl, *N*-aryl-*N*′-alkyl, and *N*,*N*′-dialkyl CDIs. (B) Competitive binding experiment to show relative stability of NHC–CDIs with *N*-aryl-*N*′-alkyl and *N*,*N*′-diaryl amidinates. (C) Control experiment testing CDI exchange for two *N*,*N*′-diaryl CDIs.

Johnson and co-workers further probed this trend by synthesizing a series of adducts containing *N*,*N*′-diaryl, *N*-aryl-*N*′-alkyl, and *N*,*N*′-dialkyl CDIs.^23,33^^1^H NMR spectroscopy was used to evaluate *K*_eq_ (Fig. 7A). As expected, *N*,*N*′-dialkyl CDIs had relatively low *K*_eq_'s, even <1 M^−1^ at room temperature for some adducts, while *N*,*N*′-diaryl CDIs gave robustly stable adducts. *N*-Aryl-*N*′-alkyl CDIs were predicted to form adducts that strike a balance between these two extremes. While the ^1^H NMR analysis showed quantitative adduct formation (*K*_eq_ ≫ 550 M^−1^) similar to *N*,*N*′-diaryl CDIs, competitive binding experiments revealed that *N*-aryl-*N*′-alkyl amidinate adducts are indeed more dynamic than their *N*,*N*′-diaryl counterparts. *N*-Aryl-*N*′-alkyl amidinates could be quantitatively displaced by *N*,*N*′-diaryl CDIs to form *N*,*N*′,*N*′′,*N*′′′-tetraaryl NHC–CDIs, with concomitant evolution of free *N*-aryl-*N*′-alkyl CDI (Fig. 7B). Monitoring this process by ^1^H NMR spectroscopy revealed a first-order rate constant for this transformation as *k*_d_ (50 °C) = (4.0 ± 0.4) × 10^−4^ s^−1^. *N*,*N*′,*N*′′,*N*′′′-Tetraaryl NHC–CDI adducts do not undergo such CDI exchange, even in the presence of a different *N*,*N*′-diaryl CDI (Fig. 7C), indicating that the adduct bond with *N*-aryl-*N*′-alkyl amidinates is dynamic at 50 °C, while the bond with *N*,*N*′-diaryl amidinates is not.

#### 3.3.2 Basicity and stability

The basicity of NHC–CDI adducts can be leveraged for the formation of (see Section 4.1) cationic amidine species *via* protonation with HCl;^26^ however, adducts with *N*,*N*′-dialkyl amidinate substituents – such as ^Me^I^i^PrCDI^iPr^ – decompose over the course of 12 h on exposure to moisture, presumably due to sufficient basicity to deprotonate water.^26^ The strong basicity of these adducts was also supported by a 1974 report^24^ on the thiazolium iodide reaction with a *N*,*N*′-diaryl CDI to form a spiro-heterocycle *via* cycloaddition. The authors' inability to isolate the proposed betaine adduct intermediate was most likely a direct result of using the weak base triethylamine to deprotonate the thiazolium salt before adding the CDI without purification. The amidinate formed upon reaction of the CDI with thiazolylidene is sufficiently basic to deprotonate the [HNEt_3_]^+^[I]^−^ in solution, even in the presence of excess triethylamine.

The dynamics of the central NHC–CDI bond also play a role in stability. While stable under inert atmospheres, NHC–CDI adducts with *N*-aryl-*N*′-alkyl amidinates are less stable to moisture compared to their *N*,*N*′-diaryl amidinate counterparts due to a more dynamic central C–C bond (see Section 3.3.1).^33^ Upon exposure to ambient conditions, the bis-adduct SIMesCDI^iPr,Ph2^ decomposes over 15 h as measured by changes in its ^1^H NMR spectra over time. Liquid chromatography mass spectrometry (LCMS) analysis of the resulting solution suggested a complex mixture of degradation products. These data indicate that while there is complete adduct formation according to ^1^H NMR spectroscopy, a small amount of free NHC is present at equilibrium, which reacts with moisture in the air, similar to ^Me^I^i^PrCDI^iPr^. Notably, no such degradation was observed for the *N*,*N*′-diaryl amidinate analogues SIMesCDI^Mes,Ph2^ and SIMesCDI^*pn*BuPh,Ph2^.

## 4. Applications

### 4.1 NHC–CDIs as ligands

**Fig. 8:**
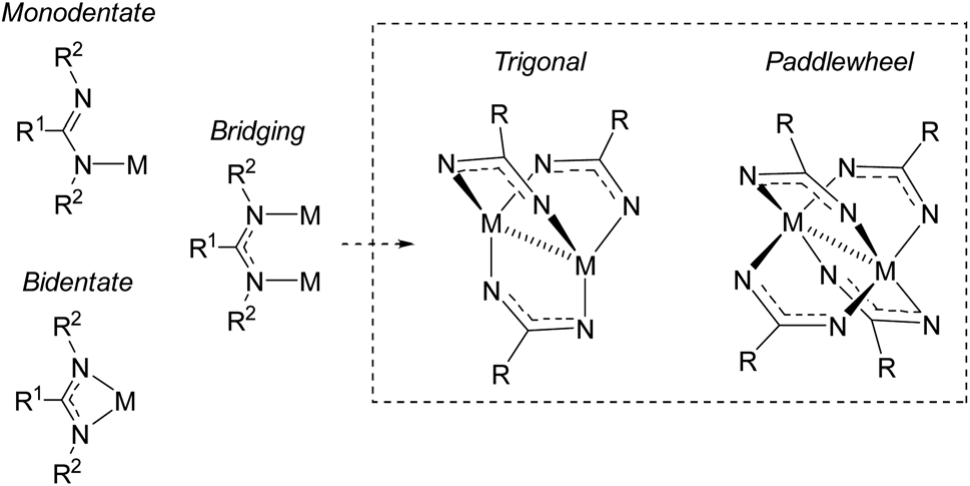
Coordination modes of amindinate ligands. R^2^ groups omitted from paddlewheel complexes for clarity.

Amidinates are π-electron rich chelating ligands, whose coordination chemistry has been extensively studied across the periodic table.^17–22^ These complexes have seen applications in homogenous catalysis^46^ (in particular, olefin polymerization^19,47^). Amidinates are the nitrogen-analogues of carboxylates, containing two nitrogen atoms that can coordinate to metals as monodentate, bidentate, or bridging ligands (Fig. 8).^46,48,49^ As bridging ligands, amidinates can generate bimetallic “paddlewheel” complexes in both trigonal and tetragonal modes.^18,50–52^ It is well known that tuning the amidinyl substituents can have a profound effect on the binding modes of these ligands.

NHC–CDI adducts can act as analogues to amidinate ligands but with an electronically neutral character due to their zwitterionic structure. This neutrality imparts bench-stability to NHC–CDIs which sets them apart from monoanionic amidinate, guanidinate, or carboxylate ligand systems that can be moisture-sensitive. Similar to amidinate ligands, the substituents at the nitrogen atoms of the N_2_C^−^ moiety of the NHC–CDI can be varied, allowing precise steric and electronic tuning of the binding site. Furthermore, the substituents at the nitrogen atoms of the N_2_C^+^ moiety can also be altered, affording further steric and electronic tuning of the ligand. This section will focus on studies of NHC–CDIs as ligands for discrete metal complexes (Section 4.1.1) and as stabilizing ligands for nanoparticle (NP) surfaces (Section 4.1.2).

#### 4.1.1 Discrete complexes

**Fig. 9:**
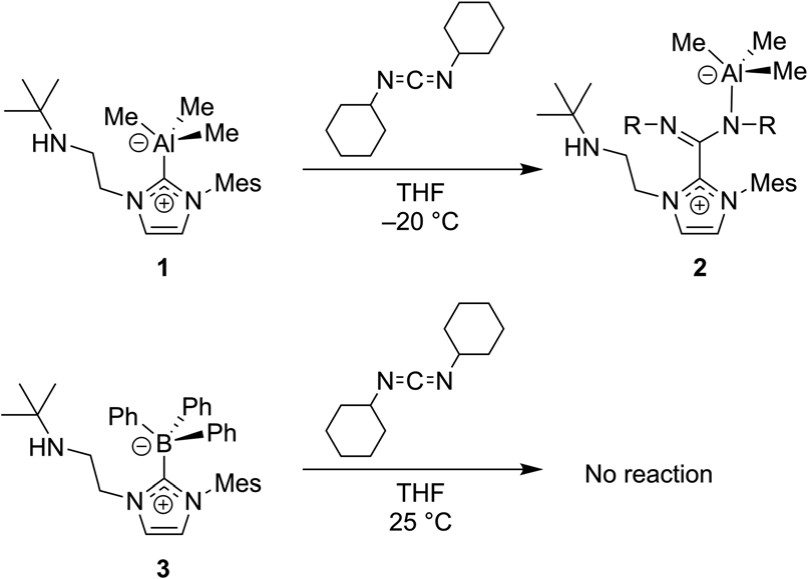
Carbodiimides such as CDI^CY^ can be used to probe the non-innocent bonding of NHCs to group 13 atoms.^53^

In 2012, Ong and co-workers reported the first NHC–CDI metal complex:^53^ an Al species bound terminally at the CDI fragment (Fig. 9). In this work they did not first isolate the NHC–CDI adduct to act as the ligand, but instead used CDI^Cy^ to probe the non-innocent bonding of group 13 atoms with NHCs. To a solution of Al–NHC species **1** in THF was added CDI^Cy^, which resulted in the formation of **2**. The solid-state structure of **2** revealed the insertion of the carbodiimide into the Al–carbene bond, yielding a zwitterion that contains aluminate and imidazolium. Measured bond distances and angles were consistent with electronic delocalization of the NHC ring. CDI^Cy^ was then reacted with boron-NHC species **3**, however insertion was not seen, illustrating the stability of the boron–carbene bond.

In 2015, Cámpora and co-workers demonstrated the first use of isolated NHC–CDI adducts as ligands, coordinated to Cu(i) metal centers.^41^ Three different NHC–CDI adducts were designed, containing either ICy or IMe as the NHC and CDI^*p*Tol^ or CDI^DiPP^ as the CDI fragment. Similar to amidinate ligands, the authors show that the steric differences between CDI^*p*Tol^ and CDI^DiPP^ can control the binding modes of the NHC–CDI adducts between terminally bound (monodentate) and bridging (Fig. 8).

**Fig. 10:**
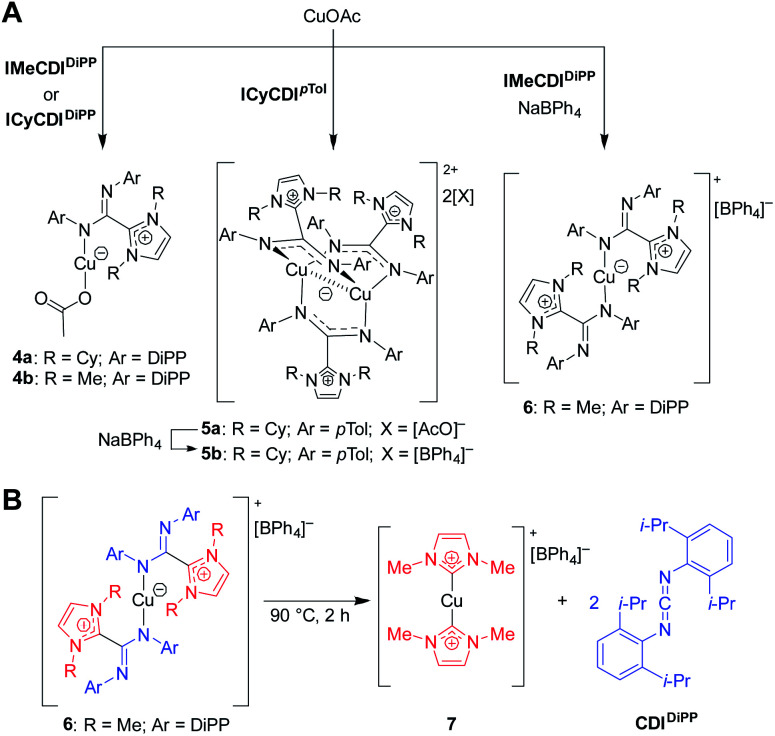
Copper complexes of NHC–CDIs.^41^ (A) Mono- and bimetallic complexes. (B) Heating complexes to 90 °C causes ejection of CDI^DiPP^ fragment and formation of a bis-NHC Cu(i) complex.

Initially, the NHC–CDI adducts were combined with copper(i) acetate with the goal of generating bridged complexes similar to those seen in mixed acetate–amidinate complexes.^54^ An equimolar mixture of Cu(i) acetate and either DiPP adduct (ICyCDI^DiPP^ or IMeCDI^DiPP^) lead to pale yellow-green 1 : 1 (CuOAc)(L) complexes, with the NHC–CDI terminally bound to the copper atom (Fig. 10A, **4a** and **4b**). The two species were characterized using single crystal XRD and NMR experiments. The ^1^H and ^13^C{^1^H} NMR signals of the NHC–CDI ligands were sharp and defined, suggesting that the monodentate coordination of the amidinate ligands was maintained in solution. The less bulky CDI^*p*Tol^ provided a multinuclear assembly. Upon exposure of ICyCDI^*p*Tol^ to CuOAc, irrespective of reagent ratio, a trigonal paddlewheel complex (**5a**) formed, consisting of two copper centers bridged by three NHC–CDI ligands. It was also found that changing the counteranion to [BPh_4_]^−^ had a negligible impact on the solid-state structure (**5b**). Unlike the yellow-green monometallic Cu(i) species discussed above, this complex exhibited an intense orange color. From the solid-state structure, a short Cu⋯Cu distance of 2.4123 Å was observed, indicating a closed shell (d^10^–d^10^) cuprophilic interaction. This interaction results in the strong, red-shifted color, with absorption bands assigned to metal–metal 3d → 4p transitions. The Cu⋯Cu bond distance is comparable to the shortest seen in binuclear Cu(i) complexes bridged by anionic amidinate or guanidinate anions (2.40–2.54 Å).^55–57^

In order to form a binuclear product similar to **5a** and **5b** with a DiPP-substituted adduct, IMeCDI^DiPP^ was exposed to CuOAc in the presence of NaBPh_4_. Regardless of whether a 1 : 1 : 1 or 2 : 3 : 2 ratio of CuOAc : IMeCDI^DiPP^ : NaBPh_4_ was used, a mononuclear [CuL_2_][BPh_4_] species was isolated (**6**), which contained two terminally-bound NHC–CDI ligands. Interestingly, upon heating a solution of the complex to 90 °C, the CDI fragments were ejected as free carbodiimide, and a bis-NHC Cu(i) complex (**7**) was formed quantitatively. This thermal decomposition also occurred for the other species discussed, with varying degrees of selectivity.

**Fig. 11:**
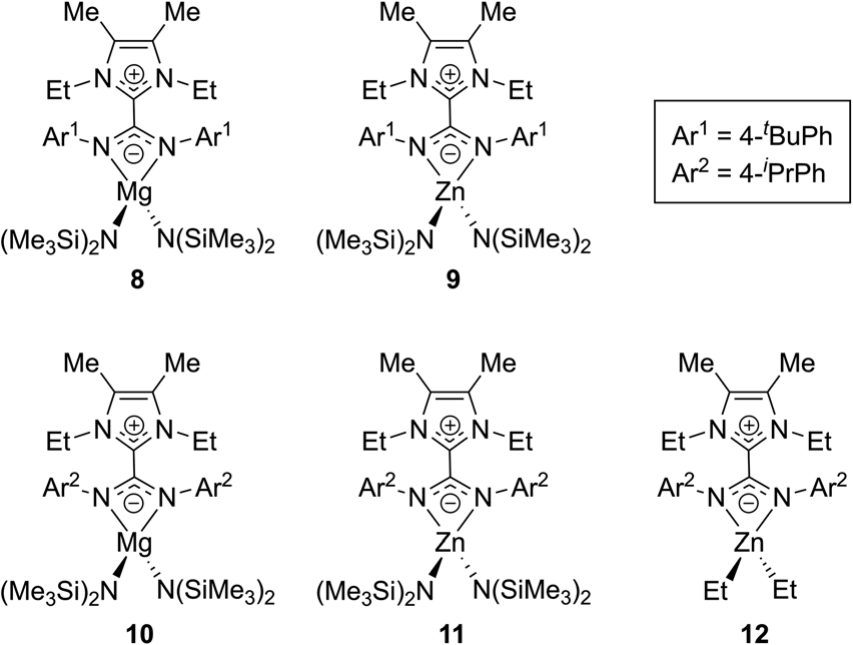
NHC–CDI complexes with Mg and Zn reported by Nembenna and co-workers.^58^

Expanding on this work, in 2017 Nembenna and co-workers isolated the first *N*,*N*′-chelated NHC–CDI adducts as magnesium and zinc complexes.^58^ Five new complexes were isolated (Fig. 11) based on previously-reported,^37^ moisture-stable NHC–CDI adducts utilizing ^Me^IEt and either 4-*tert*-butylphenyl or 4-isopropylphenyl substituents on the CDI. Initially, coordination with group 1 metals (where M = Li, Na, and K) was explored. While complexation was detected by ^1^H NMR spectroscopy, the desired products could not be isolated.

Complexes **8–11** were formed by exposure of the appropriate NHC–CDI to M{N(SiMe_3_)_2_}_2_ (M = Mg or Zn) in toluene at room temperature. Species **12** was prepared through the reaction of ^Me^IEtCDI^*p*iPrPh^ with ZnEt_2_. The complexes were characterized using multinuclear NMR experiments (^1^H, ^13^C, and ^29^Si) and single crystal XRD analysis, which all indicated *N*,*N*′-chelating binding modes for the NHC–CDIs to form four-membered rings with the metal. Ligand displacement reactions of compounds **8** and **9** were probed using neutral-type ligands (NHCs and isocyanides). No displacement of the NHC–CDI was seen, indicating a greater stability of the zwitterionic *N*,*N*′-chelate compared to neutral monodentate ligands.

Complex **8** was then tested for its utility as a carbonyl hydroboration catalyst; main-group metal amidinate complexes are known to catalyze this reaction.^59^ An equimolar ratio of 2-chlorobenzaldehyde and pinacolborane was exposed to 5 mol% of **8** in benzene-d_6_ at room temperature. Quantitative conversion of the aldehyde to the boronate ester was seen in 2 h, signifying, to our knowledge, the first use of an NHC–CDI metal complex as a catalyst.

**Fig. 12:**
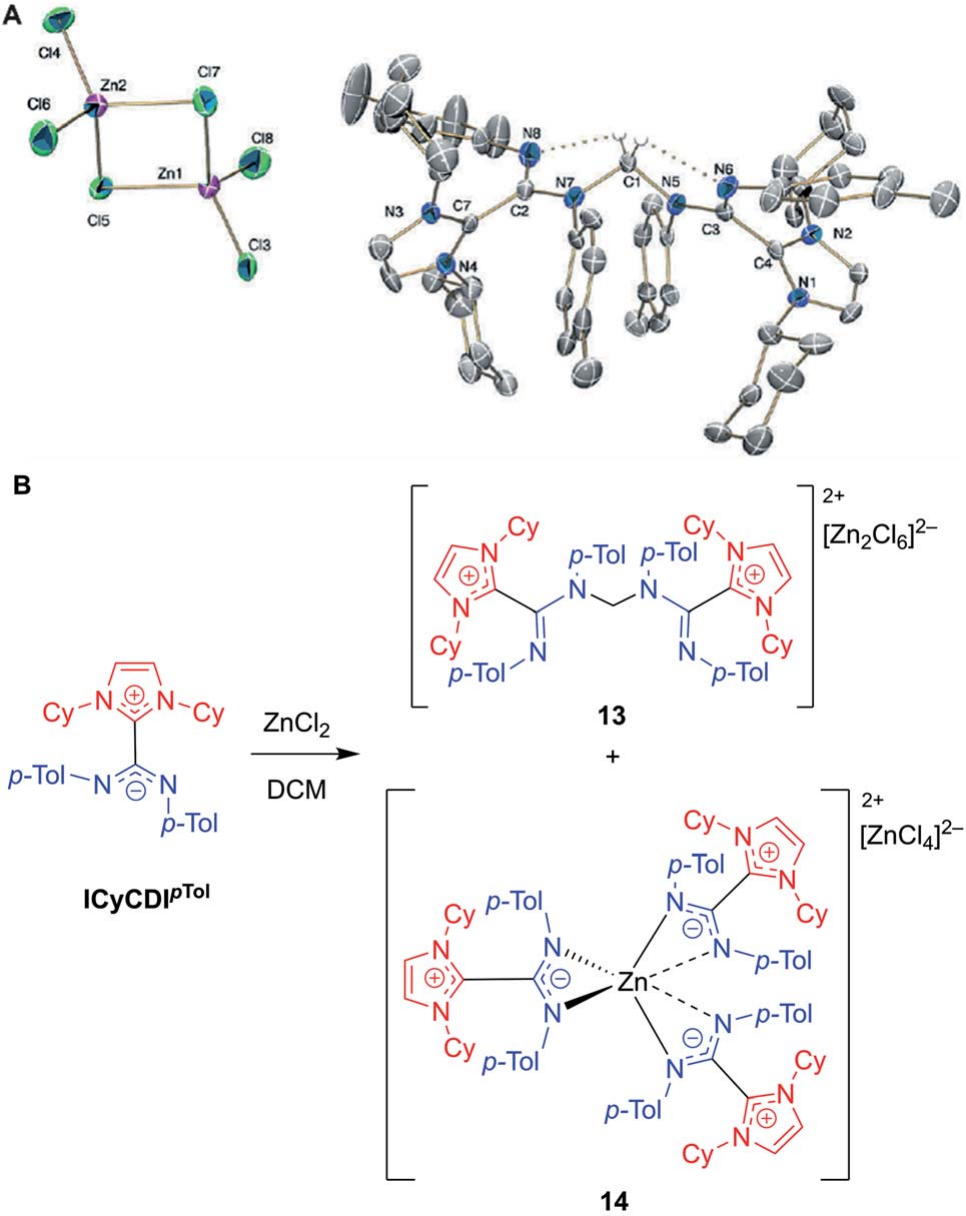
(A) Single crystal structure of methylene-bridged aminal **13**. Hydrogen bonds shown in dotted lines. Ellipsoids plotted at 30% probability. Reproduced from ref. 60 with permission from The Royal Society of Chemistry. (B) Reaction of NHC–CDI ICyCDI^*p*Tol^ with ZnCl_2_ in DCM, as reported by Cámpora and Mosquera.^60^ (Cy = cyclohexyl, *p*-Tol = 4-methylphenyl, DCM = dichloromethane).

In 2018, Cámpora, Mosquera, and co-workers reported a remarkable case of DCM activation when investigating the coordination chemistry of ICyCDI^*p*Tol^ with ZnCl_2_ (Fig. 12).^60^ Initially, the complexation reaction progressed very slowly at room temperature despite the strong basicity of the NHC–CDI adduct. After four days, the ^1^H NMR spectrum of the solution showed a large decrease in ICyCDI^*p*Tol^, accompanied by the growth of two new species. Some crystalline material was seen; however, the crystal quality was not good enough to produce a solid-state structure. In a case of serendipity while setting up an NMR experiment, ZnCl_2_ was added to a week-old CD_2_Cl_2_ solution of ICyCDI^*p*Tol^. The ^1^H NMR spectrum indicated that one of the new species was more prevalent, and crystals were grown from this solution. Single crystal XRD revealed the structure to be a methylene-bridged aminal counterbalanced by the binuclear anion [Zn_2_Cl_6_]^2−^ (**13**), suggesting that 2 equivalents of ICyCDI^*p*Tol^ underwent nucleophilic attack onto DCM in an S_N_2 fashion with concomitant loss of two equivalents of chloride. Interestingly, both amidine units, N(Ar)–C=N(Ar), adopt an *E*,*E*-configuration in **13** (Fig. 12B), while the free NHC–CDI adduct prefers a *E*,*Z*-geometry. This unusual geometry is due to intramolecular CH⋯N hydrogen bonds between the bridging methylene and CDI nitrogen atoms. In order to determine the second species seen in the ^1^H NMR spectrum, ICyCDI^*p*Tol^ was exposed to ZnCl_2_ in a 3 : 2 ratio in bromobenzene, a solvent that is less susceptible to nucleophilic attack. Through NMR and XRD studies, the species was determined to be a [ZnL_3_]^2+^ dication counterbalanced by a [ZnCl_4_]^2−^ dianion (**14**, Fig. 12A).

It was initially thought that the Lewis acidic ZnCl_2_ was assisting ICyCDI^*p*Tol^ in the nucleophilic activation of dichloromethane; however, the preferential formation of **13** from “aged” solutions of ICyCDI^*p*Tol^ suggested that the ZnCl_2_ merely acts to trap the formed cation. Monitoring a solution of ICyCDI^*p*Tol^ in CD_2_Cl_2_ over the course of a week showed slow formation of the methylene-bridged product with no observed intermediates. Additionally, ICyCDI^*p*Tol^ was shown to react with other polychloroalkane solvents, such as chloroform and 1,2-dichloroethane, eliminating HCl and generating ICyCDI^*p*Tol^H^+^˙Cl^−^, with protonation occurring at one of the amidinyl nitrogen atoms.

Due to the unusual nature of this reaction, a DFT model was built using a simplified version of the NHC–CDI adduct, using Me instead of Cy in the wingtip position. The rate-determining step was ascribed to be the initial S_N_2 attack of the adduct on dichloromethane, with a 3 kcal mol^−1^ reduction of the energy barrier if ICyCDI^*p*Tol^ rearranges from the *E*,*Z* to *E*,*E* configuration. The calculated energy barrier (28.0 kcal mol^−1^) to the first transition state was found to be in very good agreement with the experimental barrier (27.8 kcal mol^−1^). The [NHC–CDI–CH_2_Cl]^+^ intermediate then undergoes a second S_N_2 reaction with another NHC–CDI to give the bridged species. This work sets the stage for future work involving NHC–CDI adducts as nucleophiles.

#### 4.1.2 Functional nanoparticle surfaces

The synthesis of metallic nanoparticles (NPs) with controlled sizes and functional ligands has enabled numerous technological innovations in fields such as green catalysis,^61,62^ energy conversion,^63,64^ electronics,^65,66^ and biomedicine.^67,68^ Small NP size is crucial for these applications, but bare surfaces often lead to aggregation.^69^ Surface functionalization stabilizes these NPs and enables further control of the optical, electronic, and catalytic properties of the metallic core.^70,71^ NHCs have been used for the stabilization of metallic surfaces including NPs due to their ability to coordinate at the surface.^8,9,72–81^ NHC–CDIs have more recently been targeted as surface ligands; their basic nitrogen atoms may coordinate to transition metals more strongly than typical electronically neutral ligands, and their modular syntheses could open new avenues for tuning surface properties.

In 2015, Cámpora, Philippot, Chaudret, and co-workers explored the stabilization of ruthenium NPs (RuNPs) using NHC–CDIs as stabilizing ligands with different Ru : ligand ratios.^27^ It is difficult to control the size of RuNPs in the range of 1 nm, with the vast majority of NPs reported in the 1.5–1.8 nm range, depending on the stabilizer used.^82^ Metal-to-ligand ratio is commonly used to control NP size, with increased ligand density typically leading to smaller NPs. Thus, the authors hypothesized that the large steric protection afforded by NHC–CDIs could lead to smaller NPs.

**Fig. 13:**
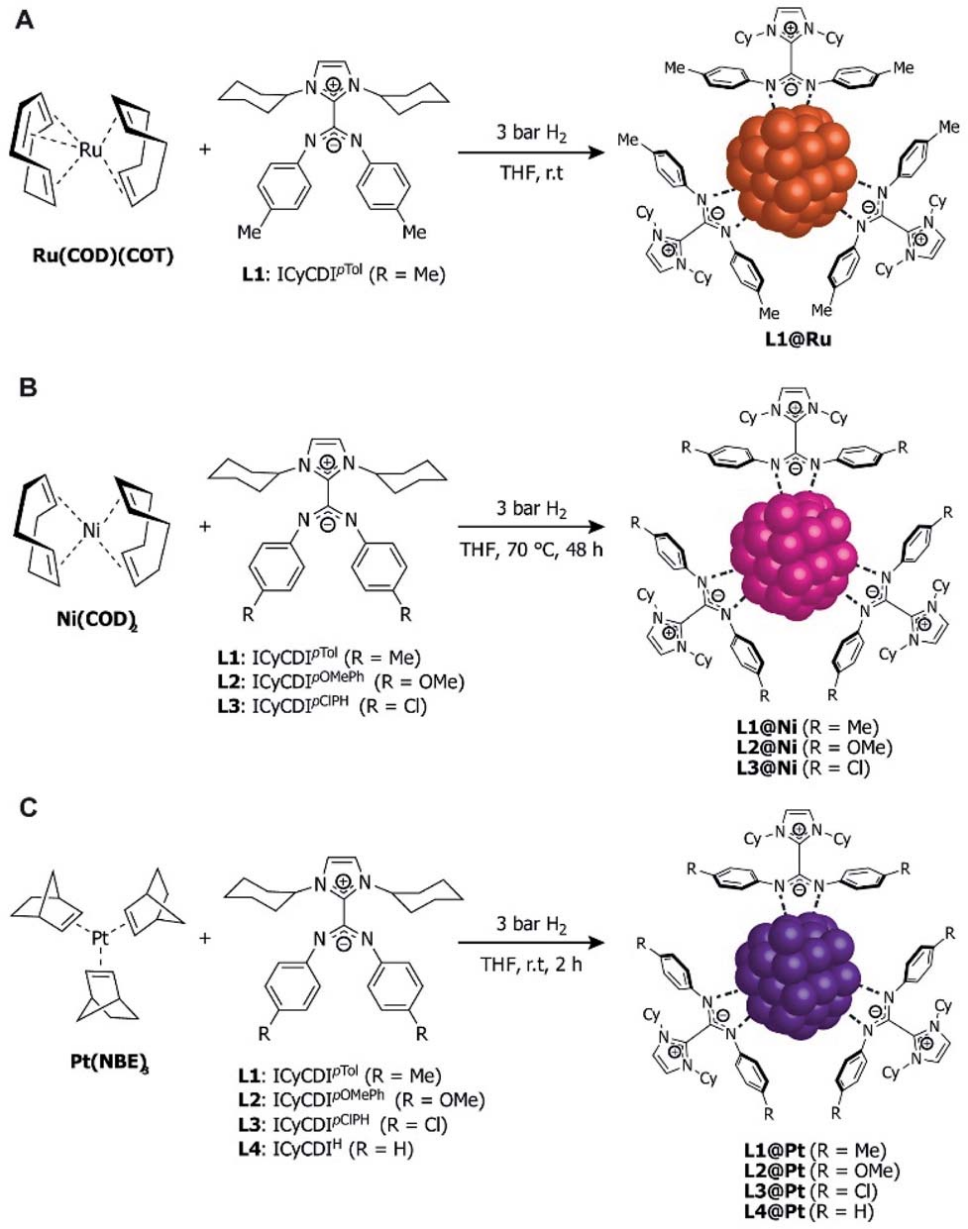
Synthesis of NHC–CDI-stabilized nanoparticles. (A) Ru(COD)(COT) to form RuNPs,^27^ (B) Ni(COD)_2_ to form NiNPs,^42^ and (C) Pt(NBE)_3_ to form PtNPS.^30^

Toward this end, ICyCDI^*p*Tol^ was added to THF solutions of Ru(COD)(COT) (COD = 1,5-cyclooctadiene, COT = 1,3,5-cyclooctatriene) at room temperature under 3 bar H_2_ (Fig. 13A) with ligand : Ru ratios of 0.1, 0.2 and 0.5. **L1(0.1)@Ru**, **L1(0.2)@Ru**, and **L1(0.5)@Ru** had mean particle diameters of 1.3 nm, 1.0 nm, and 1.0 nm, respectively, as determined by transmission electron microscopy (TEM) and radial distribution function (RDF) analysis. Increasing the ligand from 0.1 to 0.2 equiv. resulted in the expected NP size reduction, but a further increase to 0.5 equiv. had no effect on the RuNP diameter due to steric hindrance.^83^

To test the coordination stability of the NHC–CDI ligands, **L1(0.1)@Ru** was exposed to 10 equiv. of octanethiol and the mixture was heated at 65 °C in C_6_D_6_ for 24 h. ^1^H and ^13^C{^1^H} NMR spectroscopy did not show the presence of free NHC–CDI in the solution, indicating that, assuming free NHC–CDI would be stable under the reactions conditions, the strongly coordinating thiol was unable to displace the ligand to a discernible amount on the NMR timescale. Di-*n*-octyl disulfide, however, was observed, due to the catalytic oxidation of the octanethiol at the metal surface, suggesting that free Ru sites are still accessible as further confirmed by CO binding experiments.

Metal nanoparticles are attractive for catalysis because the facile tuning of both the electronic configuration and surface area of the particle combine the advantages of homogenous and heterogenous catalysts.^84^ They frequently display high catalytic activities and can often be reclaimed at the end of the reaction. RuNPs are known to catalyze hydrogenation of alkenes and arenes under mild conditions; thus, **L1(0.1)@Ru** and **L1(0.2)@Ru** were tested for their performance in styrene hydrogenation to probe the selectivity between vinyl and aromatic moieties. With **L1(0.1)@Ru**, full hydrogenation to ethylcyclohexane was observed after 24 h, while **L1(0.2)@Ru** gave a mixture of 26 : 74 ethylbenzene and ethylcyclohexane over the same time period. Therefore, **L1(0.2)@Ru** has greater selectively for vinyl hydrogenation over **L1(0.1)@Ru**, attributed to fewer faces present in **L1(0.2)@Ru** compared to **L1(0.1)@Ru**.

In 2020, Martínez-Prieto, van Leeuwen, and co-workers prepared nickel nanoparticles (NiNPs) stabilized by three different NHC–CDI ligands^42^ featuring ICy and *para*-substituted phenyl groups at the amidinyl nitrogens (R = Me, OMe, Cl for **L1**, **L2** and **L3**, respectively). NiNPs were formed by exposing Ni(COD)_2_ to 0.2 equiv. of the appropriate ligand in THF at 70 °C under H_2_ at 3 bar (Fig. 13B). The resultant spherical **L1@Ni** and **L2@Ni** NPs had sizes of ∼2.8 nm, while **L3@Ni** NPs were larger (3.4 nm) and considerably more disperse. Structural characterizations were performed using high-resolution TEM, wide-angle X-ray scattering (WAXS), powder X-ray diffraction (pXRD), and X-ray photoelectron spectroscopy (XPS).

In order to screen the catalytic properties of the NiNPs, **L1@Ni** was tested in the hydrogenation of 8 substrates containing different functional groups (alkenes, alkynes, and carbonyl groups). The hydrogenation reactions proceeded in toluene at room temperature and utilized 3 mol% Ni. Double and triple carbon–carbon bonds both underwent hydrogenation, with 3-hexyne and diphenylacetylene displaying high selectivities for the (*Z*)-alkenes and only traces of the (*E*)-alkenes and corresponding alkanes. Carbonyls were not hydrogenated to a significant extent under the same conditions.

Electronic variations at the amidinate moiety (R = Me, OMe, Cl) were tested in the context of 3-hexyne hydrogenation in toluene. At long reaction times (8 h), all three species showed >99% conversion, with selectivity (78–90%) to produce the (*Z*)-olefin. At shorter reaction times (2–6 h), lower conversions were seen, with the more electron-withdrawing species **L3@Ni** (R = Cl) displaying the lowest activity. **L3@Ni** was, however, found to have a slightly higher selectivity towards the formation of (*Z*)-3-hexene, possibly due to its lower activity. The catalytic activity of these NiNPs are higher than those of other Ni-based systems previously reported.^85^ Notably, the NiNPs could be easily removed from solution with a magnet and recycled up to 3 times while maintaining good activity.

In 2020, Cámpora, van Leeuwen, and co-workers^30^ utilized the modularity of NHC–CDIs to synthesize Pt nanoparticles (PtNPs) of varying diameters in order to experimentally probe the effect of NP size on the Knight shift, which is a feature in the NMR frequency of a paramagnetic species caused by an interaction between nuclear spins and the spins of conduction electrons.^86,87^ Knight shifts occur for metal NPs due to the presence of free electrons on the surface; they can complicate the NMR chemical shifts of both the metallic isotope and the ligands bound to the surface. “Ultra-small” (<1 nm) Pt nanoparticles, however, do not exhibit a Knight shift due to the absence of free electrons on the surface and, thus, behave similarly to molecular species.^88^

The PtNPs were stabilized using similar NHC–CDI adducts, consisting of ICy as the NHC fragment and *para*-substituted phenyl groups on the CDI nitrogens (R = Me, OMe, Cl, H for **L1**, **L2**, **L3** and **L4**, respectively).^27,41^ PtNPs were formed by reaction of Pt(NBE)_3_ (NBE = 2-norbornene) in THF under 3 bar H_2_ with the appropriate NHC–CDI adduct (Fig. 13C). Pt(NBE)_3_ is advantageous over typical precursors, such as Pt_2_(DBA)_3_ (DBA = dibenzylideneacetone) or Pt(CH_3_)_2_(COD), due to faster reaction times and the ability to remove norbornene under vacuum. The PtNPs were synthesized with differing **L1** : Pt ratios of 0.1, 0.2 and 0.5 molar equiv. (**L1(0.1)@Pt**, **L1(0.2)@Pt**, and **L1(0.5)@Pt**), giving particle diameters of 2.3 nm, 2.1 nm, and 1.9 nm, respectively, as determined by TEM. Crystallinity was confirmed using WAXS. As expected, elemental analysis (EA) indicated that the NHC–CDI adducts remained intact on the NP surface. FTIR studies comparing the strong stretching C=N bands of the free adduct **L1** at 1530 and 1495 cm^−1^ and the particle **L1(0.5)@Pt** at 1630 and 1598 cm^−1^ suggested that the ligand binds through the amidinate moiety.

Surface properties of the PtNPs were probed using solid state NMR spectroscopy by exposing the nanoparticles to ^13^CO and then obtaining the ^13^C MAS NMR spectrum to monitor the Knight shift. The paramagnetic effect caused by surface electrons of the NP cause the ^13^C signals of the CO to be shifted downfield and broadened considerably (the Knight shift). As the size of the NP is decreased from 2.3 nm to 1.9 nm, a small decrease in the magnitude of the Knight shift was observed. Regardless, in all cases, the large, broad Knight-shifted CO signals (∼100–600 ppm, centered ∼400 ppm) obscure any other resonances in the spectrum. As a correlation was seen between NP size and the magnitude of the Knight shift on the CO band, the authors prepared PtNPs of average ∼1.2 nm diameter using Pt_2_(DBA)_3_ as the platinum source in a ligand : Pt ratio of 0.2 molar equiv. in order to suppress the Knight-shifted signal. Following exposure to ^13^CO, the ^13^C MAS NMR showed three clear CO resonances between 190–230 ppm. The Knight-shifted CO resonance is heavily suppressed, though still present due to CO coordinated to larger PtNPs present in the sample. Additional FTIR studies showed that CO is adsorbed to the Pt surface in both bridging and terminal coordination modes.

It is important to note here the similarities in NP synthesis between the three reports discussed (Fig. 13). All NHC–CDI adducts were synthesized using methods discussed in 3.1.3 and differ only in the substituent at the *para*-position of the phenyl groups attached at the CDI nitrogens. Irrespective of the metal precursor used, all NPs were formed by reacting the metal complex with the NHC–CDI in THF under 3 bar H_2_ and purified by precipitation with pentane. This straightforward methodology, coupled with the subtle structural and electronic effects afforded by NHC–CDIs, allows for fine-tuning of NP size and catalytic activity and may find utility in metal nanoparticle and metal nanocluster applications at the frontier of molecular and solid-state chemistry.

### 4.2 NHC–CDIs in zwitterionic materials

Zwitterionic polymers comprise macromolecules that contain both cationic and anionic groups either along the main chain or incorporated into the side chain. These polymers have gained interest across a variety of fields due to their biocompatibility, antifouling properties, high ionic conductivity, and ability to stabilize nanoparticles and proteins.^89^ Zwitterionic polymers can be prepared from mixtures of oppositely charged monomers or from zwitterionic monomers.^89,90^ The vast majority of the latter materials are made with pendant sulfobetaine, carboxybetaine, or phosphobetaine moieties,^89–91^ though main-chain polybetaines have also been synthesized from phospholipids,^92,93^ polysquaraines,^94^ and NHC-isothiocyanate adducts.^95^

**Fig. 14:**
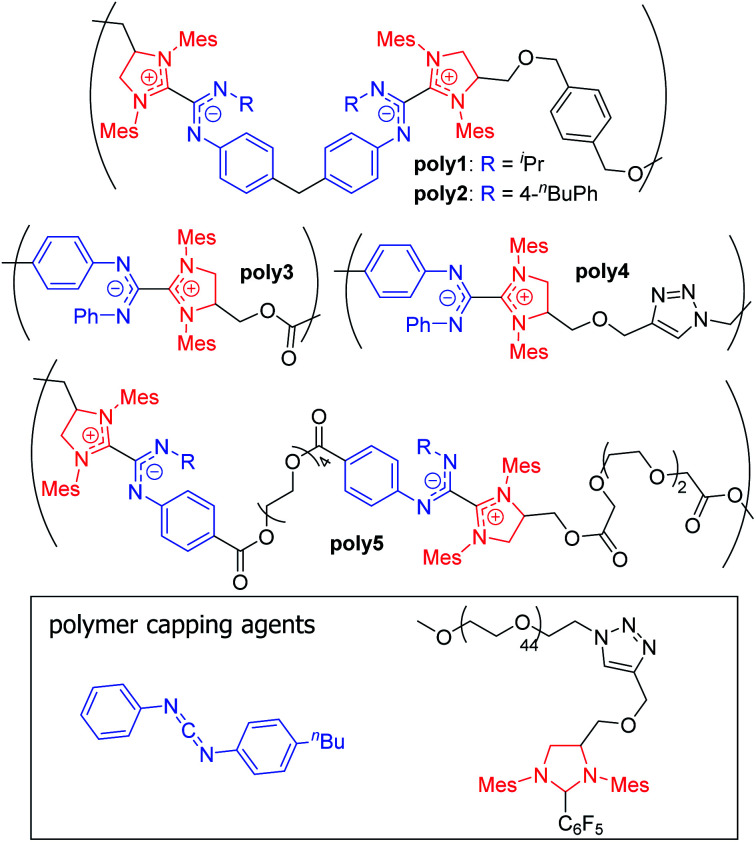
Structures of previously synthesized poly(azolium amidinates) (PAzAms).^33^ (Mes = 2,4,6-trimethylphenyl).

In 2018, Johnson and co-workers reported a new class of polybetaines based on NHC–CDI adducts called poly(azolium amidinate)s (PAzAms, Fig. 14).^33^ The dative-character of the NHC–CDI adduct bond (see Section 3.3.1) makes PAzAms supramolecular polymers where the equilibrium of adduct formation (*K*_eq_) and rate of dissociation (*k*_d_) will affect the material properties. NHC–CDIs are good candidates for making such supramolecular polymers due to their high modularity leading to control over *K*_eq_, *k*_d_, adduct geometry, charge delocalization, air- and moisture-stability, and the potential to use the resulting amidinate-type fragments for metal ion ligation.

Initially, two polymers were made *via* the step-growth polymerization of bis-imidazolidinium salts and bis-CDIs (AA + BB type monomers, **poly1** and **poly2** in Fig. 14). On the basis of different adduct stabilities in small molecule NHC–CDIs (see Section 3.3.1), PAzAms were synthesized from both bis-(*N*-aryl-*N*′-alkyl CDI)s and bis-(*N*,*N*′-diaryl CDI)s. Number-average molar masses (*M*_n_) of ∼14.0 kDa for **poly1** and ∼6.5 kDa for **poly2** were obtained by ^1^H NMR spectroscopy, which corresponds to degrees of polymerization (DP) of ∼11 and ∼4, respectively. Gel permeation chromatography (GPC) traces exhibited broad dispersities (*Đ*) expected from a step-growth polymerization. It was found that the preparative procedure for these polymers greatly affected their molar masses; filtering the salt byproduct after deprotonation of the imidazolidinium salt resulted in low DP (DP_NMR_ = 2–4) for both polymers, perhaps due to considerable loss of the bis-NHC during filtration. Removing this filtration step resulted in higher DP for both **poly1** (DP_NMR_ = 11) and **poly2** (DP_NMR_ = 9), however, the GPC trace of **poly2** was irregular and displayed a very broad dispersity (*Đ* = 2.87), likely due to aggregative effects. When bis-NHC pentafluorobenzene adducts were used as the monomer, DP = 8 **poly2** was isolated and displayed a more typical GPC trace, but the polymer was not very soluble in THF. Therefore, the lower DP oligomers of **poly2** formed using the filtration procedure were used for initial investigations.

The dynamics of **poly1** imparted by the *N*-aryl-*N*′-alkyl CDI were probed in many ways. First, depolymerization was instigated through heating to 50 °C in the presence of a free *N*,*N*′-diaryl CDI (CDI^*p*Tol^). First-order kinetics suggested that this process is rate-limited by NHC–CDI adduct dissociation followed by rapid trapping of the free carbene by *N*,*N*′-diaryl CDI. Second, **poly1** could be converted to **poly2** in the presence of CDI^*p*BuPh,Ph2^ at 50 °C for 12 h. Complete CDI exchange occurs as the thermally-labile **poly1** is converted to the stable **poly2**. Finally, the stability of **poly1** to ambient conditions was tested. While **poly1** seems indefinitely stable in solution under inert atmosphere, significant decomposition upon exposure to air was documented *via*^1^H NMR spectroscopy, GPC, and LCMS over the course of 18–24 hours. This was explained *via* degradation of the small fraction of free NHCs present at equilibrium. In contrast, **poly2** was very stable to both depolymerization and ambient conditions. Depolymerization could be achieved, but only at 100 °C in molten CDI^*p*Tol^.

The air- and moisture-stability of **poly2** prompted further exploration of *N*,*N*′-diaryl CDIs in the context of supramolecular polymers. Heat-activated pentafluorophenyl-masked NHCs were designed to obviate the need for deprotonation of imidazolidinium salts by strong base (see Section 3.1.1). Both AA + BB type and AB type monomers were synthesized, showing the power of this methodology to access both pairwise alternating (+ + − −) and purely alternating (+ −) charge sequences. Monofunctional end caps were utilized to control the molecular weight of the PAzAms and/or introduce end functionality to the chains. Due to perfect 1 : 1 stoichiometry, AB monomers facilitated higher DP polymers of ≥25 for **poly3** and ∼16 for **poly4**. As expected, increasing the amount of chain-capping agent leads to progressively lower DPs. The DP for AA + BB oligomers could be controlled *via* monomer ratio, varying between 2 and 8. Generally, hydrophobic linkers led to higher DPs compared to polymers with hydrophilic linkers (**poly5**), possibly due to residual moisture in hydrophilic monomers leading to unwanted termination events. As expected, all *N*,*N*′-diaryl CDI-derived PAzAms displayed excellent stability under ambient conditions, which was tested by heating to 70 °C in 1,2-dichloroethane-*d*_4_ under air. The solid material could be annealed at 100 °C for 24 h under N_2_ with no significant change in *M*_n_; however, in the presence of free CDI^*p*Tol^, depolymerization takes place under these conditions, underscoring the supramolecular nature of these polymers. Thermogravimetric analysis (TGA) also supports high thermal stability, as mass loss was not observed until ∼175–200 °C.

Most of the reported PAzAms were quite hydrophobic; in order to test the stability of these polybetaines in aqueous environments, a polyethyleneglycol (PEG)-containing capping agent was utilized to increase water solubility. The ratio of hydrophilic to hydrophobic blocks was controlled by the amount of capping agent added. The following samples were prepared: PEG2k-*b*-(AzAm)_4_, PEG2k-*b*-(AzAm)_8_, and PEG2k-*b*-(AzAm)_25_ (AzAm = the repeat unit of **poly3**) and all exhibited good water solubility (5–30 mg mL^−1^). ^1^H NMR spectroscopy in D_2_O showed evidence of aggregation-induced peak broadening as expected for amphiphilic block copolymers;^96^ however, the material could be extracted back into organic solvents without showing any significant changes compared to the native sample. These data indicate that PAzAms are stable in aqueous environments for at least several hours at room temperature. The aggregation of these materials was further investigated using dynamic light scattering (DLS) and TEM after dialysis into water from acetone. DLS showed a Gaussian size distributions and average particle diameters ranging from 66–100 nm; no correlation between particle size and hydrophobic block size was observed, perhaps due to the high dispersities (1.47–1.85) of the materials. TEM revealed that most of the aggregates had spherical morphologies of similar sizes to those measured by DLS except for a small amount of elongated worm-like morphologies for PEG2k-*b*-(AzAm)_8_. This work lays the foundation for future materials and tunable surface modification based on PAzAm zwitterionic polymers. Recently, Larsen and co-workers were inspired by these dynamic bonds to develop thermal guanidine-based covalent adaptable networks.^97^

### 4.3 Distonic radical cation stabilization

Radicals play critical roles in biological and chemical processes, and efforts to harness the reactivity of organic radicals has led to applications in organic synthesis,^98,99^ energy storage,^100,101^ biological imaging,^102,103^ and in polymerization reactions.^104,105^ It is well studied in the literature that carbenes – in particular NHCs – have been used to stabilize a wide range of main-group radicals^106,107^ due to their strong σ-donating and π-accepting properties. These species include boryl,^108^ silyl,^109^ pnictogenyl,^110,111^ and aluminium-bound adducts.^112^ Aminyl radicals that have been stabilized by NHCs^113,114^ were extensively delocalized into the empty p-orbital of the carbene. Through leveraging the unique electronic properties of neutral, zwitterionic NHC–CDIs, Johnson and co-workers investigated an alternative mode of radical stabilization that does not rely on the π-accepting ability of the carbene in the hopes that these stabilized radical species could be used for a range of redox-active materials.^44^

**Fig. 15:**
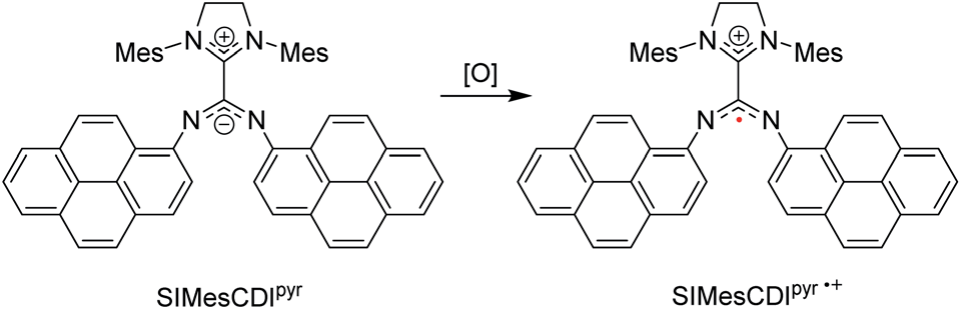
NHC–CDI distonic radical cation reported by Johnson and co-workers.^45^ (Mes = 2,4,6-trimethylphenyl, [O] = oxidant).

In this work, pyrene-substituted SIMesCDI^pyr^ is oxidized to generate a “distonic” radical cation (SIMesCDI^pyr^˙^+^), *i.e.*, a radical cation where the charge and spin occupy spatially distinct regions within the same molecule (Fig. 15). The positive charge resides on the NHC moiety, while the unpaired electron is stabilized across the *N*-pyrene substituents. As a comparison, analogous phenyl-derived amidinate species were also synthesized; however, as expected, the generated radical cations of these compounds were too reactive to be observed at room temperature due to insufficient resonance delocalization, causing the compounds to quickly degrade.

The electrochemical properties of the species were studied using cyclic voltammetry (CV). The SIMesCDI^pyr^ showed a reversible one-electron oxidation, corresponding to the distonic radical cation. The phenyl species, however, displayed irreversible oxidation in their CVs, suggesting a stabilizing influence of the pyrene moieties. Chemical oxidation of the SIMesCDI^pyr^ with silver bistriflimide (AgTFSI) also formed the distonic radical cation, and the extensive delocalization of the unpaired electron across the pyrene groups was observed using electron paramagnetic resonance (EPR) spectroscopy. The EPR spectrum was simulated with the unpaired electron coupled to the two amidinyl nitrogens, which was in good agreement with experimental data and consistent with *C*_2_ symmetry in solution. Coupling to 10 protons—corresponding to the bound pyrene groups—was observed, with a significant spin density across each π-system. DFT calculations supported the assignment of a distonic radical cation, with the spin density and singly occupied molecular orbital (SOMO) occupying only the amidinate and pyrene groups on the adduct, with minor contributions from the cationic imidazolidinium moiety. The increased resonance stabilization afforded by the pyrene substituents allowed the distonic radical cation to exist even after exposure to air, with no significant change in the EPR spectral intensity observed. The phenyl-substituted analogues with reduced π-systems, however, showed dimerization after chemical oxidation.

Redox applications are a potential use for this new type of persistent, distonic radical cation. To demonstrate an example, the authors used SIMesCDI^pyr^ as a catholyte (*i.e.*, the electrolyte on the cathode side of an electrochemical cell) in an organic redox-flow battery, with *N*-methylphthalimide (*N*-MPI) as the anolyte. During the first charging cycle, the distonic radical showed a flat voltage plateau around 150 mV *vs.* Ag/Ag^+^. The species also demonstrated a high reversibility, with an average coulombic efficiency of 94.6% after 20 charging cycles and no performance decay for 100 cycles.

## 5. Conclusions and outlook

This *Perspective* is a comprehensive survey of the nascent field of NHC–CDI betaine adducts. While hypothesized as far back as the 1970s, it has only been the recent discovery of robust, air- and moisture-stable variants based on *N*,*N*′-diaryl CDIs that has sparked the rapid growth in this field. The highly modular nature of both NHCs and CDIs has enabled the synthesis of many adducts with electronic and steric modifications that allow the probing of structure–property relationships. Although all currently known adducts utilize imidazol-2-ylidene and imidazolidin-2-ylidene NHCs, other NHC classes – such as 1,2,4-triazol-3-ylidene, thiazol-2-ylidene, and cyclic alkylaminocarbenes – could result in even greater variety of properties and applications in the future.

Coordination complexes containing NHC–CDIs as net-neutral amidinate-like ligands have been synthesized with terminal, chelating, and bridging binding modes. They can also stabilize a variety of metal nanoparticles. A few of these examples showed catalytic behavior, but given the breadth of reactions traditional amidinate metal complexes can catalyze^17,19^ – including hydroamination,^115^ olefin polymerization,^116^ and lactone polymerization^117^ – it seems inevitable that NHC–CDIs will become more popular in the future as versatile, electroneutral ligands for catalysts. The basicity that makes these adducts good ligands also contributes to their nucleophilicity, as shown by the reaction of ICyCDI^*p*Tol^ with DCM, hinting at the promise of appending these moieties to organic molecules. Building on these efforts and work done on zwitterionic polymers, NHC–CDIs present vast opportunities for nanoparticle and surface functionalization in the future. These modifications could be done with either functional small molecules or through the polymerization of PAzAms from a surface. Designing hydrophilic monomers for these supramolecular polymers will open up new regimes to explore, such as antibiofouling and biological properties. An example of an application that has already been realized is the bis(pyrene) NHC–CDI that could stabilize a distonic radical to be used as a catholyte in a redox flow battery.

Beyond the fields presented here, there is a remarkable opportunity for NHC–CDIs in the field of organocatalysis. The strong precedence of NHC betaine adducts as latent organocatalysts for Umpolung reactions or lactone polymerization poise NHC–CDI adducts as a more modular alternative to the common carboxylate adducts. It is clear that the NHC–CDI scaffold holds a lot of promise across a variety of fields and the positive attributes of facile synthesis, modularity, and robustness provide a desirable toolbox for exciting research for many years to come.

## Conflicts of interest

The authors have no conflicts to declare.
